# *Aristolochia Manshuriensis* Kom Inhibits Adipocyte Differentiation by Regulation of ERK1/2 and Akt Pathway

**DOI:** 10.1371/journal.pone.0049530

**Published:** 2012-11-14

**Authors:** Dong Hoon Kwak, Ji-Hye Lee, Taesoo Kim, Hyo Sun Ahn, Won-Kyung Cho, Hyunil Ha, Youn-Hwan Hwang, Jin Yeul Ma

**Affiliations:** Traditional Korean Medicines (TKM)-Based Herbal Drug Research, Herbal Medicine Research Division, Korea Institute of Oriental Medicine, Daejeon, Republic of Korea; University of Cincinnati, United States of America

## Abstract

*Aristolochia manshuriensis Kom* (AMK) is a traditional medicinal herb used for the treatment of arthritis, rheumatism, hepatitis, and anti-obesity. Because of nephrotoxicity and carcinogenicity of AMK, there are no pharmacological reports on anti-obesity potential of AMK. Here, we showed AMK has an inhibitory effect on adipocyte differentiation of 3T3-L1 cells along with significantly decrease in the lipid accumulation by downregulating several adipocyte-specific transcription factors including peroxisome proliferation-activity receptor γ (PPAR-γ), CCAAT/enhancer binding protein α (C/EBP-α) and C/EBP-β, which are critical for adipogenesis *in vitro*. AMK also markedly activated the extracellular signal-regulated protein kinase 1/2 (ERK1/2) pathway including Ras, Raf1, and mitogen-activated protein kinase kinase 1 (MEK1), and significantly suppressed Akt pathway by inhibition of phosphoinositide-dependent kinase 1 (PDK1). Aristolochic acid (AA) and ethyl acetate (EtOAc) fraction of AMK with AA were significantly inhibited TG accumulation, and regulated two pathway (ERK1/2 and Akt) during adipocyte differentiation, and was not due to its cytotoxicity. These two pathways were upstream of PPAR-γ and C/EBPα in the adipogenesis. In addition, gene expressions of secreting factors such as fatty acid synthase (FAS), adiponectin, lipopreotein lipase (LPL), and aP2 were significantly inhibited by treatment of AMK during adipogenesis. We used the high-fat diet (HFD)-induced obesity mouse model to determine the inhibitory effects of AMK on obesity. Oral administration of AMK (62.5 mg/kg/day) significantly decreased the fat tissue weight, total cholesterol (TC), and low density lipoprotein-cholesterol (LDL-C) concentration in the blood. The results of this study suggested that AMK inhibited lipid accumulation by the down-regulation of the major transcription factors of the adipogensis pathway including PPAR-γ and C/EBP-α through regulation of Akt pathway and ERK 1/2 pathway in 3T3-L1 adipocytes and HFD-induced obesity mice, and AA may be main act in inhibitory effects of AMK during adipocyte differentiation.

## Introduction

Obesity is a major risk factor for a number of metabolic diseases including cardiovascular diseases, hypertension, and diabetes mellitus (type 2) [1], [2]. Obesity is an increase in the number of differentiated mature adipocytes and a condition in which excess body fat has accumulated due to lipids becoming adipocytes [3]–[5]. Adipogenesis, the differentiation process that produces adipocytes, is a complex process that involves various changes including gene expression, hormone sensitivity, and cellular morphology [6], [7]. Commonly *in vitro* model system for adipogensis is used the 3T3-L1 cells which originally derived from mouse embryos [8]. Differentiation of 3T3-L1 preadipocytes into mature adipocytes was induced by up stimulation with 3-isobutyl-1-methylxanthine (IBX), dexamethasone (Dex) and insulin, and was promoted the accumulating large amount of intracellular lipid droplets in mature adipocytes [9]. In the process of adipogenesis, it is well-known that peroxisome proliferation-activity receptor-γ (PPAR-γ) and CCAAT/enhancer binding protein-α (C/EBP-α) are master regulators, [6], [10] which leads to induce the expression of lipid metabolizing enzymes such as fatty acid binding protein (FABP) 4 and lipoprotein lipase (LPL) [7]. In studies of 3T3-L1 cells show C/EBP-ββ and C/EBP-α δ are expressed before and activate the transcription of these master regulators PPAR-γ and C/EBP-α. Several studies have been reported that extracellular signal-regulated kinases (ERK) and Akt pathway play essential roles in adipogenesis [11]–[13]. From these studies showed that ERK1/2 phosphorylation had an important role in early stage of adipocytes differentiation [14]. Furthermore, the inhibitory effect of epigallocatechin gallate and genistein on adipocyte differentiation was mediated by ERK1/2 pathway [15], [16]. Insulin is an important proadipogenic hormone, requires the activation of both Akt and ERK1/2 for adipogenesis [17], [18]. The involvement of Akt in adipocyte differentiation is more established and inhibition of Akt pathway has been shown to reduction of adipogenesis [19], [20].

World-wild for thousands of years, have been used medical plants for therapy of diseases [21]. *Aristolochia manshuriensis* Kom (AMK), a perennial shrub belonging to the *aristolochiaceae* family, is distributed throughout northeastern countries such as China, Korea, and Japan [22]. It species are traditional Chinese medicine (TCM) used as analgesic, antibacterial, anti-inflammatory, antitussive, and anti-asthmatic agents as well as for the treatment of snake bites [23]. Although the major constitute of AMK species, aristolochic acid (AA) was reported the leads to serious side effects such as nephrotoxicity and carcinogenicity [24]. Some TCM doctors still suggest that these natural medicines can be used in specific ways, including the ingestion of low doses over a short period of time and external use as anti-inflammation and antibacterial agents.

Since, there are no reported that pharmacological reports on anti-obesity potential of AMK to date. Thus, we used 3T3-L1 adipocytes to test the anti-obesity effects ability of AMK. Therefore, in present study, we investigated the anti-obesity effects of AMK extracts in adipogenesis of 3T3-L1 preadipocytes and high-fat diet (HFD)-induced obesity mouse model.

## Results

### Effects of AMK Extract on 3T3-L1 Differentiation and Cell Viability

**Figure 1:**
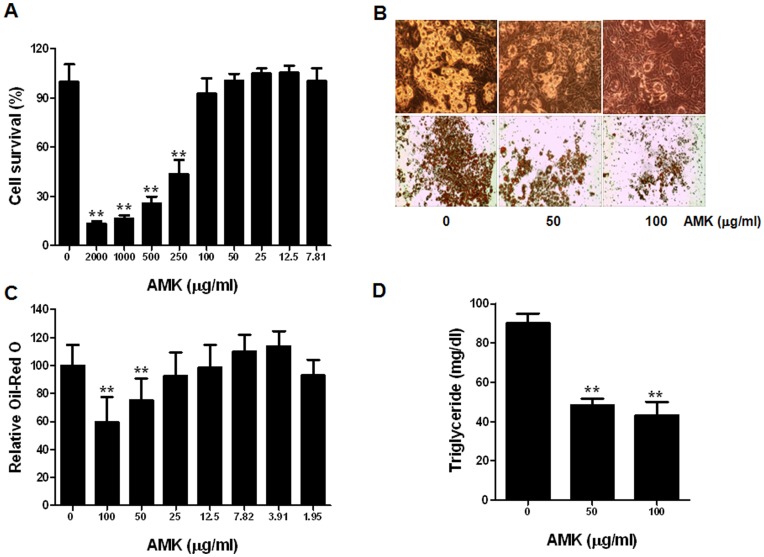
Extract of AMK inhibits adipocyte differentiation of 3T3-L1 cells. Two-day post-confluent 3T3-L1 preadipocytes (day 0) were treated with the indicated concentrations of *aristolochia manshuriensis* Kom extract and was repleted every 2 days along with relevant media cocktail up to day 8. Cells treated with 1X PBS were used as control. (A) Cell viability was determined by MTT assay. (B) Intracellular lipids were stained Oil-Red O. (C) Absorbance was spectrophotometrically determined at 500 nm after Oil-Red O staining. (D) Triglyceride (TG) content (per mg protein) was measured with a TG-S reaction kit (Asan Pharm. Co., Seoul, Korea). Three biological replicates of *aristolochia manshuriensis* Kom extract-treated adipocytes were tested. The results were confirmed by three independent experiments, which were each conducted in triplicate. Data are expressed as the mean ± S.D. **P<0.01 vs. controls.

To investigate the effect of AMK extract on adipocyte differentiation, we examined the accumulation of intracellular lipid. Confluent 3T3-L1 cells treated with DM containing Dex, IBMX and insulin. 3T3-L1 cells were induced to differentiate to adipocytes by DM, resulting in significant accumulations of intracellular lipid droplets. These lipid droplets were stained using Oil-Red O dye as shown in Figure 1B. The lipid droplet accumulation was significantly reduced following treatment with 50 or 100 µg/mL of AMK extract (Figure 1C). The main component of lipid accumulation in adipocytes is TG. When cellular TG contents were measured, TG levels were markedly increased during *in vitro* adipogensis of 3T3-L1 cells. Following the addition of 50 and 100 µg/mL of AMK extract into the medium and TG accumulation was also significantly inhibited compared with control (Figure 1D). Viability of 3T3-L1 cells treated as described above, measured using MTT assay, and was not significantly affected at concentration down of 100 µg/mL (Figure 1A). These results indicate that the inhibitory effect of AMK extract on TG accumulation was not due to its cytotoxicity. The inhibitory effect of AMK extract on *in vitro* TG accumulation was evident at a concentration of as little as 50 µg/mL of AMK extract (Figure 1C and D).

### Extract of AMK Inhibits the mRNA Level of Transcription Factors and Adipocyte Markers in Adipogensis

**Figure 2:**
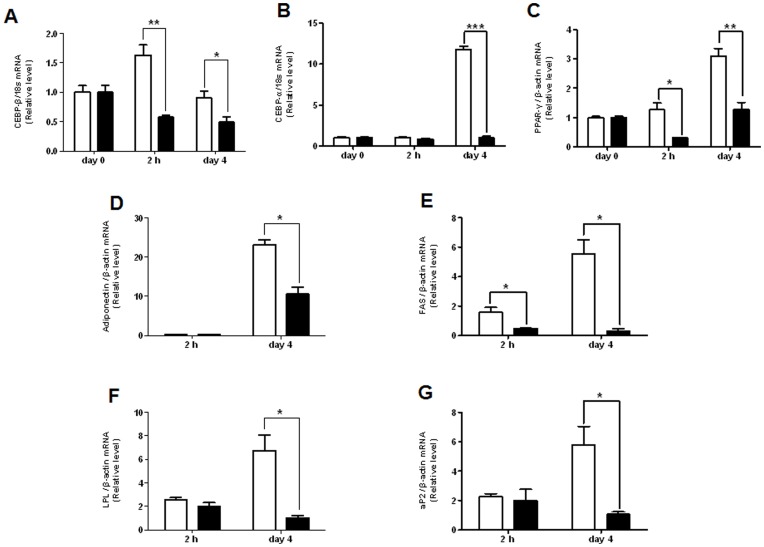
Effect of AMK extract on the expression of transcription factors and adipocyte-specific genes in differentiation of 3T3-L1 cells. Preadipocytes were induced to differentiate with extract of *aristolochia manshuriensis* Kom (100 µg/mL) and harvest at 2 h and day 4 during the differentiation period. (A) The mRNA of C/EBP-β. (B) The mRNA of C/EBP-α. (C) The mRNA of PPAR-γ. (D) The mRNA of adiponectin. (E) The mRNA of FAS. (F) The mRNA of LPL. (G) The mRNA of aP2. The mRNA was analyzed by real-time PCR. Results were expressed relative to untreated cells after normalization to 18s rRNA and β-actin mRNA. Values are mean ± S.D. of data from three separate experiments; each experiment was performed in triplicate. *P<0.05 and **P<0.01 vs. control. White bars are a control without the extract of AMK, and black bars are with the extract of AMK (100 µg/mL).

Two major transcription factors, PPAR-γ and C/EBP-α, have been reported to regulate a large part of gene expression involved in adipogenesis [10]. PPAR-γ, a transcription factor of the nuclear-receptor superfamily, and C/EBP-α, a member C/EBP family basic-leucine zipper class of transcription factors, expression increased during differentiation of 3T3-L1 cells. These induced expressions of two major transcription factors were regulated by C/EBP-β and C/EBP-α. In this study, it was investigated that the mRNA expression of PPAR-γ and C/EBP family (C/EBP-α and C/EBP-β) in adipogenesis of 3T3-L1 cells were treated with extract of AMK (Figure 2). The C/EBP-β was limited to the early phase of differentiation and was completely inhibited the increase of C/EBP-β mRNA levels by AMK extract (Figure 2A). The level of C/EBP-α mRNA was significantly decreased in induction of adipogenesis after 4 days (Figure 2B). The extract treatment significantly reduced the amounts of PPAR-γ mRNA (Figure 2C). PPARs are key transcription factors for adipogensis and lipogenensis overexpression of PPAR-γ induces adipocyte differentiation of 3T3-L1 cells. On the other hand, suppression of PPAR-γ expression blocks adipogenesis and lipogenesis. PPARs mediate the transcription of a group of genes related to fatty acid synthesis, oxidation, transport, storage, or energy expenditure. Extract of AMK down-regulated the expression of C/EBP-α and PPAR-γ. We investigated that the expression of their target genes including, aP2, LPL, adiponectin, and FAS might also be down-regulated. The mRNA levels of aP2, LPL, adiponectin, and FAS in 3T3-L1 adipocyte differentiation were almost completely suppressed by AMK extract (Figure 2D–G). This result suggested that extract of AMK inhibits adipogenesis through reducing the expression of C/EBP-β which leads to down- regulate the expression of C/EBP-α and PPAR-γ.

### Extract of AMK Inhibits the Differentiation of 3T3-L1 Cells by Regulation of ERK1/2 Pathway and Akt Pathway

**Figure 3:**
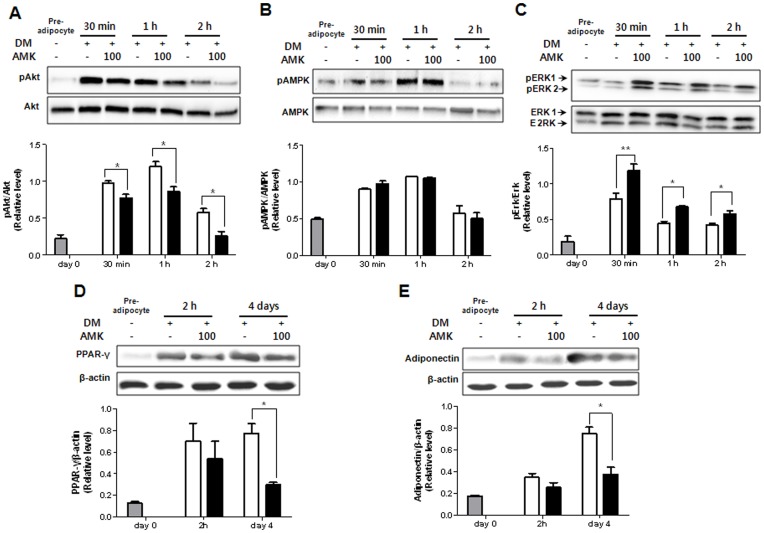
Extract of AMK inhibits the differentiation of 3T3-L1 cells by regulation of ERK1/2 phosphorylation and Akt phosphorylation. Preadipocytes were induced to differentiate with extract AMK (100 µg/mL) and harvest at 30 min, 1 h and 2 h and during the early-stage of differentiation. (A) The expression of Akt. (B) The expression of AMPK. (C) The expression of ERK1/2. Preadipocytes were induced to differentiate with extract of AMK (100 µg/mL) and harvest at 2 h and day 4 during the differentiation period. (D) The expression of PPAR-γ as a major factor for adipogenesis. (E) The expression of adiponectin as an adipocytes-specific factor. The proteins were analyzed by western blot. Results were expressed relative to untreated cells after normalization to β-actin mRNA. Values are mean ± S.D. of data from three separate experiments; each experiment was performed in triplicate. *P<0.05 and **P<0.01 vs. control. Gray bars are a 3T3-L1 preadipocyte, white bar is a 3T3-L1 differentiation without the extract of AMK, and black bars are 3T3-L1 differentiation with the extract of AMK (100 µg/mL).

**Figure 4:**
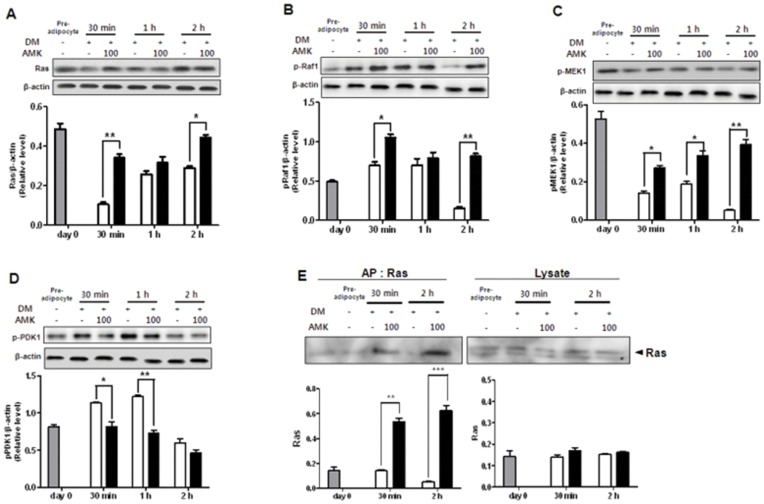
Extract of AMK regulates the upstream of ERK1/2 pathway and Akt pathway during early stage of 3T3-L1 adipocyte differentiation. Preadipocytes were induced to differentiate with extract of AMK (100 µg/mL) and harvest at 30 min, 1 h and 2 h and during the early-stage of differentiation. (A) The expression of Ras. (B) The expression of pRaf1. (C) The expression of pMEK1. (D) The expression of pPDK1. (E) The assay of membrane-Ras activation. The proteins were analyzed by western blot. Results were expressed relative to untreated cells after normalization to β-actin mRNA. Values are mean ± S.D. of data from three separate experiments; each experiment was performed in triplicate. *P<0.05, **P<0.01 and ***P<0.001 vs. control. Gray bars are 3T3-L1 preadipocyte, white bars are a 3T3-L1 differentiation without the extract of AMK, and black bars are 3T3-L1 differentiation with the extract AMK (100 µg/mL).

**Figure 5:**
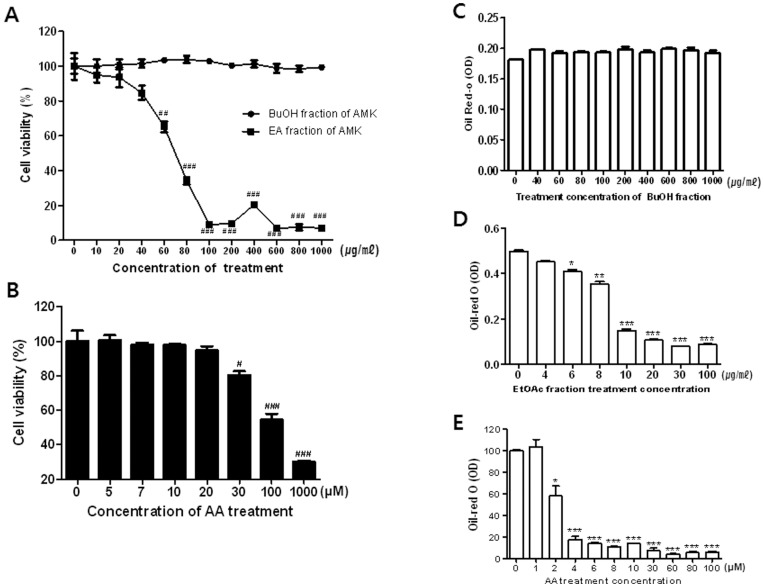
AA and fractions of AMK constitutes inhibits adipocyte differentiation of 3T3-L1 cells. Two-day post-confluent 3T3-L1 preadipocytes (day 0) were treated with the indicated concentrations of AA and fractions of AMK constitutes such as EtOAc fraction with AA and BuOH fraction without AA, and was repleted every 2 days along with relevant media cocktail up to day 8. Cells treated with 1X PBS were used as control. (A) Cell viability was treated with fractions of AMK constitutes (EtOAc and BuOH) and (B) AA was determined by MTT assay. Intracellular lipids were stained Oil-Red O. (C) Absorbance was spectrophotometrically determined at 500 nm after Oil-Red O staining (C) treatment of BuOH fraction; (D) treatment of EtOAc fraction; (E) treatment of AA. The results were confirmed by three independent experiments, which were each conducted in triplicate. Data are expressed as the mean ± S.D. **P<0.01 vs. controls.

**Figure 6:**
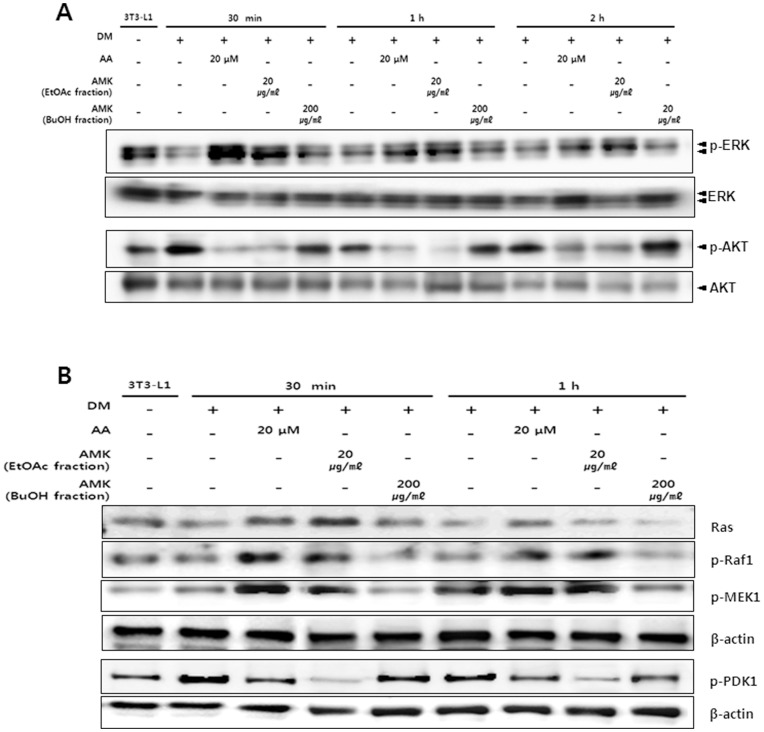
AA and fractions of AMK constitutes inhibits the differentiation of 3T3-L1 cells by regulation of Ras/Raf/MEK/ERK1/2 phosphorylation and PDK/Akt phosphorylation. Preadipocytes were induced to differentiate with AA (20 µmol), EtOAc fraction (20 µg/mL), BuOH fraction (200 µg/mL), and harvest at 30 min, 1 h and 2 h and during the early-stage of differentiation. (A) The phosphorylation of ERK1/2 and Akt. (B) The expression of Ras, pRaf1, pMEK1, and pPDK1. The proteins were analyzed by western blot. Results were expressed relative to untreated cells after normalization to β-actin mRNA. Values are mean ± S.D. of data from three separate experiments; each experiment was performed in triplicate.

It has been reported that ERK1/2 and Akt pathways are critical for controlling adipogenesis. In the insulin signaling pathway, Akt and ERK1/2 are upstream of adipocyte differentiation pathways including PPAR-γ and C/EBP-α pathway. Therefore, in this study, to evaluate the effect of AMK extracts on upstream signaling pathway of PPAR-γ and C/EBP-α, we investigated the effects of AMK extract on the levels of phosphorylated Akt and phosphorylated ERK1/2. In 3T3-L1 preadipocytes, the phosphorylation of ERK1/2 and Akt were both significantly activated during the early stage of adipogenesis, and the activation continued as far as 3 h after induction of adipocyte differentiation by DM. The treatment of AMK extract significantly inhibited the phosphorylation of Akt (21%; *P*<0.01 at 30 min, 38%; *P*<0.05 at 1 h and 55%; *P*<0.05 at 2 h) compared with control (Figure 3A). However, phosphorylation of ERK1/2 (34%; *P*<0.05 at 30 min, 34%; *P*<0.05 at 1 h and 28%; *P*<0.05 at 2 h) was increased compared with control (Figure 3C). While extract of AMK showed less or even no phosphorylation of AMPK and the total contents of ERK1/2 and Akt (Figure 3B). Akt pathway has been shown to regulate adipocyte differentiation by modulation of PPAR-γ expression. The extract treatment abrogates PPAR-γ protein expression in adipogenesis (Figure 3D). The target gene of PPAR-γ as well as adiponectin, protein expression was significantly decreased compared with control (Figure 3E). The results suggested that the inhibition of adipocyte differentiation by extract of AMK was associated with the regulation of ERK1/2 and Akt phosphorylation. In addition, in this study, to evaluate the effect of AMK extracts on upstream signaling pathway of ERK1/2 and Akt, we investigated the effects of AMK on up-streams of ERK1/2 including mitogen-activated protein kinase kinase 1 (MEK1), Raf1 and Ras. In 3T3-L1 adipocyte differentiation, the treatment of AMK extract significantly activated the MEK1, Raf1 and Ras during early adipogensis (Figure 4A–C). We were exanimated the Ras activation using Ras activation assay kit. The Ras activation was significantly increased by AMK. Therefore the results suggested that anti-adipogenesis of AMK extract was occurred by directly increase of membrane-Ras activation of ERK1/2 pathway. While activation of phosphoinositide-dependent kinase 1 (PDK1), as a main mediator up-stream of Akt phosphorylation was significantly inhibited compared with control during adipogenesis early stage (Figure 4 E). In addition, we investigated the PIP3 expression, however PIP3 expression similar with control (Data not view). This result indicated that inhibition of Akt pathway was directly occurred by inhibition of PDK1 phosphorylation. The results suggested that the inhibition of adipocyte differentiation by extract of AMK was associated with the regulation of ERK1/2 and Akt up-streams. In present study, we investigated the TG accumulation and viability of adipocytes was treated with AA and fractions of AMK such as ethyl acetate (EtOAc) fraction with AA, and Buthanol (BuOH) fractions without AA. In this study finds that TG accumulation was significantly reduced at concentration from 6 µg/mL to 20 µg/mL of ethyl acetate EtOAc fraction of AMK extracts, cell viability was not significantly affected at concentration down to 20 µg/mL (Figure 5A and D). However, BuOH fractions of AMK extracts were not markedly changed at all concentration (Figure 5A and C). Moreover, intracellular lipid accumulation was significantly inhibited during 3T3-L1 differentiation was treated with concentration from 20 µmol to 2 µmol of AA, cell viability was not markedly changed at doses down to 20 µmol of AA (Figure 5B and E). These results were evident the inhibitory effects of AMK extract during differentiation of 3T3-L1 preadipocytes was related with AA. In addition, we investigated the effects of AA and fractions of AMK such as EtOAc fraction with AA, and BuOH fractions without AA on Ras/Raf1/MEK1/ERK1/2 pathway and PDK1/Akt pathway during differentiation of 3T3-L1 preadipocytes. In this study finds that treatment of AA and EtOAc fraction were significantly activated the phosphorylation of ERK1/2 and Akt during early of adipocyte differentiation, respectively (Figure 6A). Moreover, Ras/Raf1/MEK1 expression was strongly increased and PDK1 expression markedly inhibited during 3T3-L1 differentiation was treated with AA or EtOAc fraction of AMK, respectively (Figure 6B). However, treatment of BuOH fraction was no significantly affected Ras/Raf1/MEK1/ERK1/2 pathway and PDK1/Akt pathway during adipocyte differentiation (Figure 6). These results indicated that AMK extract inhibited the two pathways (Ras/Raf1/MEK1/ERK1/2 pathway and PDK1/Akt pathway) during adipocytes differentiation by AA as a major constitute of AMK.

### Anti-effects of AMK Extract on HFD-induced Obesity Mouse Model

**Figure 7:**
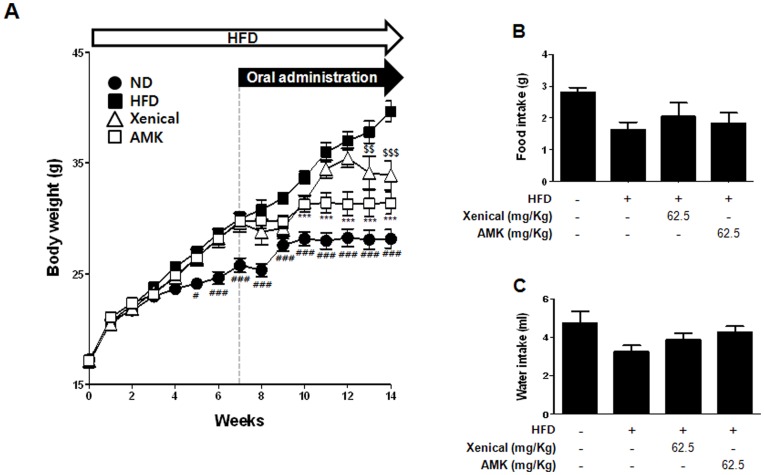
Effects of AMK Extract in HFD-induced obesity mice. Mice (n = 8 per group) were orally administrated with vehicle or extract of AMK (62.5 mg/kg/day) with HFD for 8 weeks. Normal diet (ND) fed mice were administrated with vehicle. Xenical (62.5 mg/kg/day) was orally administrated as a positive control. (A) Changes in body weight. (B) Food intake and (C) water intake of normal group, HFD-induced obsity group, positive group, and treatment of AMK extract group. Each bar represents mean ± S.D. form eight mice. ^#^ *P*<0.05 vs. HFD+vehicle, ^##^ *P*<0.01 vs. HFD+vehicle, ^###^ *P*<0.001 vs. HFD+vehicle, ***P<0.001 vs. HFD+vehicle, ^$$^ *P*<0.01 vs. HFD+vehicle and ^$$$^ *P*<0.001 vs. HFD+vehicle.

**Figure 8:**
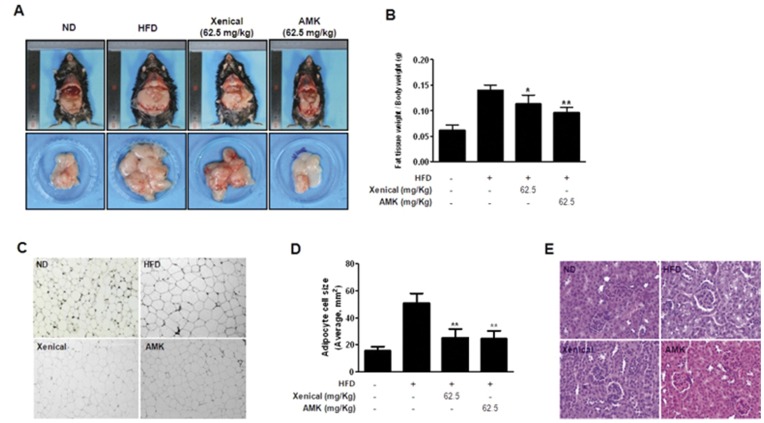
Extract of AMK inhibits the fat accumulation in HFD-induced obesity mice. Mice (n = 8 per group) were orally administrated with vehicle or extract of AMK (62.5 mg/kg/day) with HFD for 8 weeks. Normal control group was administrated normal diet (ND) fed with vehicle. Negative control group was administrated HFD with vehicle. Positive control group was administrated HFD with xenical (62.5 mg/kg/day). (A) Morphology of changed fat tissues. (B) Comparison of fat pad weight from abdominal subcutaneous and perrenal white adipose tissues. (C) Histological analysis of the adipose tissues. (C) Panel a: normal control, panel b: negative control, panel c: positive control and panel d: treatment of AMK extract. (D) Comparison of adipocyte cells size (average size, mm^2^). (E) H&E staining of kidney tissue. Sections were stained with hematoxylin and eosin dye, and using a light microscope. Each bar represents mean ± S.D. form fat tissues of eight mice. *P<0.05 vs. negative control and **P<0.01 vs. negative control.

We examined the effects of AMK extract on metabolic abnormality in HFD-fed mice. There are two types of obesity animal models available. One includes genetic models such as the leptin-deficient *ob/ob* mouse model and the leptin receptor deficient *db/db* mouse model. The other type includes the hyperlipidemia model. In this study, we used the HFD-induced obesity model to determine the inhibitory effects of AMK extract on obesity since obesity is an experimental model with much higher applicability to human obesity. To examine the effect of reducing the body weight by AMK, mice were fed HFD for 7 weeks before administration with AMK or xenical. After inducing the obesity, mice were subdivided into four groups: the normal diet group with vehicle treatment, the 60% HFD group with vehicle treatment as a negative control, the 60% HFD group with xenical (62.5 mg/kg/day) treatment as a positive control, the 60% HFD group with AMK (62.5 mg/kg/day). After inducing obesity with HFD for 7 weeks, there were significant differences in body weight between the HFD and normal diet (Figure 7A). The body weight of mice group administered with extract of AMK (62.5 mg/kg/day) was lower by 21% (*P*<0.001) as a compared with the vehicle-treated HFD group (Figure 7A). The level of daily food intakes was unchanged, suggesting that the anti-obesity effects of AMK extract were not mediated by a reduction of food and water intake (Figure 7B and C). When the extract of AMK was administered orally (62.5 mg/kg/day) to HFD mice for 8 weeks, fat tissue weight were significant reduction as much as 30% - 40% (*P*<0.01) compared with the HFD group (Figure 8A and B). In H&E stain of fat tissues, adipocyte cells sizes were small compared with HFD group (Figure 8C and D). Serum low density lipoprotein-cholesterol (LDL-C) and total cholesterol (TC) level were also significantly reduced following extract of AMK treatment (Table 1). The kidney function of mice group administered with extract of AMK (62.5 mg/kg/day) was normal (Figure 8E).

**Table 1:** Effects of AMK extract in HFD-induced obesity mice.

Measurement	ND	HFD	Xenical	AMK
			(62.5 mg/kg)	(62.5 mg/kg)
SGOP (IU/L)	152.9±44.8	169.33±71.71	183.3±94.36	203.8±101
SGPT (IU/L)	22.33±6.96	70.67±48.93#	35.78±10.79*	37.78±7.58
UREA (mg/dl)	1.44±0.3	1.53±0.32	1.47±0.17	1.31±0.35
GLUCOSE(mg/dl)	224.2±45.26	272.7±59.58	240.4±27.6	145.1±22.34
TG (mg/dl)	92.22±19.61	93.11±22.7	89±16.97	82.22±18.83
TC (mg/dl)	124.2±21.27	215.6±42.42##	160.7±29.95**	181.33±12.73*
HDL-C (mg/dl)	111.8±17.79	160.4±18.38##	142.3±20.15	158.4±12.88
LDL-C (mg/dl)	11.88±2.15	21.78±5.92##	11.78±0.94**	15.04±1.63**

Data are means ± SD (n = 9).

# *P*<0.05 and.

## *P*<0.01 indicates statistically significant differences when compared with control group.

* *P*<0.05 and.

** *P*<0.01 indicates statistically significant differences when compared with HFD group.

## Discussion

In the past several years, plant extracts have been verified as a beneficial form of medication with less potentially hazardous side effects. The anti-obesity effects of many plant extracts are reported to be mediated by adipogenesis regulation. Previous studies were reported that many natural compounds including genistein, esculetin, berberine, resveratrol, guggulsterone, conjugated linoleic acid, capsaicin, baicalein, and procyanidins inhibited adipogenesis [25], [26]. These results of previous studies suggest that various natural compounds inhibit adipogensis. In this study, experimental results demonstrate that extract of AMK significantly inhibited 3T3-L1 preadipocytes differentiation in relation to the regulation of ERK1/2 and Akt pathway.

Insulin signaling pathway plays an essential role in 3T3-L1 adipocyte differentiation [27]. In insulin signaling pathway, two downstream kinases, phosphorylation of ERK and Akt have an important roles in 3T3-L1 adipocyte differentiation. The role of ERK during adipocyte differentiation is based on the precise time of ERK phosphorylation in regulating adipogenesis [28]. Several studies have reported that inhibition of ERK during early-stages of adipogensis, as an upstream signaling of PPAR-γ and C/EBPs, induced adipogensis by activating the factors that regulated PPAR-γ and C/EBPs expression [17], [29], [30]. Other studies showed that activation of Akt could induce the differentiation of 3T3-L1 adipocytes [19], [31] and that Akt phosphorylation was inhibited in anti-adipogenesis [32], [33]. In addition, in this study, to evaluate the effect of AMK extracts on up-stream signaling pathway of ERK1/2 and Akt, we find that the extract of AMK activated the up-stream mediators of ERK1/2 including MEK, Raf and Ras (Figure 4A–C). The ERK1/2 was regulated by MEK that is the main mediator downstream of Raf, and the Raf is a main mediator downstream of Ras [34]. The membrane-Ras activation was significantly increased by AMK extract (Figure 4E). While activation of PDK1 was inhibited compared with control during adipogenesis early stage (Figure 4D). In previously study, reported that PDK1 is an up-stream kinase that phosphorylates and activates Akt [35]. In addition, we investigated the expression of PIP3 as an up-stream of PDK1, however PIP3 expression similar with control (Data not view). Therefore, results of this study, suggested that extract of AMK-induced inhibition of PPAR-γ, C/EBP-α and C/EBP-β expression might be related to the regulation of Ras activation in ERK1/2 pathway and PDK1 activation in Akt pathway. Some studies reported that AA is a major constitute of AMK, initiates apoptosis, trans-differentiation, and necrosis in kidney [36]. However, in this study, we not observed the pathogenesis such as apoptosis, and necrosis in kidney of diet-induced obesity (DIO) animals was treated with AMK (Figure 8E). Cell toxicity was not significantly increased, and TG accumulation was markedly reduced during adipocyte differentiation with treatment of AA and EtOAc fraction of AMK, respectively (Figure 5). In addition, this results show that AA and EtOAc fraction with AA, significantly regulated the Ras/Raf/MEK/ERK1/2 pathway and PDK/Akt pathway during adipogenesis, and treatment of BuOH fraction without AA was similar to control (Figure 6). In present study, results suggested that the effects of AMK in TG droplet accumulation and regulation of two early pathways (ERK1/2 and Akt) were induced by AA of AMK constitutes, and was not due to its cytotoxicity during adipocyte differentiation cells.

Adipogenesis is highly regulated by two primary adipogenic transcription factors, PPAR-γ and C/EBPs [37]. Among these, PPAR-γ is well known as master adipogenic transcription [10]. The expression of PPAR-γ is lead to induce adipogenesis in mesenchyme stem cells and fibroblasts [38], [39]. In previous studies of adipogenesis, reported that regulators of adipogenesis regulated to adipogenesis by regulating of PPAR-γ expression [7]. PPAR-γ is also known to binding to C/EBP-α promoter region that induce the expression of C/EBP-α [40]. C/EBP-α is a transcription factor of the C/EBP family basic-leucine zipper class, and was regulated by C/EBP-β in adipocyte differentiation [41]. C/EBP-β is rapidly and transiently expressed, and is required for mitotic clonal expansion (MCE) in early-stages of induced differentiation by DM [42]. The temporally expressed C/EBP-β is activated by cAMP and act to induce the expression of C/EBP-α and PPAR-γ [6]. Results of this study indicate that extract of AMK during adipogensis considerably reduces the mRNA level of C/EBP-β (Figure 2A), and that it significantly inhibited the expression of C/EBP-α and PPAR-γ genes (Figure 2B and C). Recently studies have been reported that inhibition of adipocyte differentiation by down-regulation of C/EBP-α and PPAR-γ with affecting the expression of upstream regulators C/EBP-β [39], [42]. Therefore, extract of AMK meaningfully suppressed the expression of C/EBP-β, C/EBP-α, and PPAR-γ genes in 3T3-L1 preadipocytes differentiation. These results are clearly suggested that extract of AMK inhibited the adipogensis through reduced the expression of C/EBP-α and PPAR-γ genes occurs dependently of C/EBP-β gene expression. C/EBP-α is well-known to form a positive feedback loop with PPARγ to strengthen the expression of adipocyte-specific genes [37], [43], [44], including LPL, adiponectin, FAS and aP2. In previous study, was reported that the PPAR-γ protein binds to the promoter regions of adipocyte-expressed genes, including LPL [45]. In our study, AMK was able to repress expression of PPAR-γ target genes adiponectin, aP2, FAS and LPL (Figure 2D–G). The results of this study suggested that extract of AMK down-regulates the expression of C/EBP-α and PPAR-γ, leading to decreased expression of aP2, LPL, adiponectin and FAS.

*In vivo*, we used a HFD to induce obesity model in mice [46]. The mice in obesity groups had higher body weight, total cholesterol (TC), TG, and low density lipoprotein-cholesterol (LDL-C) levels compared to normal control group (Table 1). These results indicated that the obesity mice model with higher body weight and TG, TC, and LDL-C accumulation was induced successfully by HFD. Body weights of HFD-induced obesity mice were monitored after daily oral administration of AMK extract 62.5 mg/kg/day for 7 weeks. An interesting finding in the *in vivo* study was that extract of it caused decrease body weight (Figure 7A), fat tissues weight (Figure 8A and B) and adipocyte cell size (Figure 8C and D) in obesity mice. However, it did not affect food and water intake (Figure 7B and C). AMK was decreased TC and LDL-C concentration in the blood of obesity mice (Table 1). These results are supported by our *in vitro* study that demonstrated that extract of AMK decreased lipid accumulation by inhibition of C/EBPα and PPARγ expression through regulation of Akt and ERK1/2 pathway in early stage of 3T3-L1 differentiation.

The results of this study suggested that extract of AMK inhibited adipocyte differentiation of 3T3-L1 and reduced the body weight and the fat accumulation in HFD-induced obesity mice by AA of AMK constitutes. It’s anti-obesity mechanism involves the down-regulation of the major transcription factors of the adipogensis pathway including PPAR-γ and C/EBP-α by regulation of Akt and ERK1/2 pathway, and resultant down-regulation of lipid metabolizing enzymes, FAS, LPL and aP2 which are involved in the transport, uptake and synthesis of lipids needed for the accumulation of lipid in adipocytes.

## Materials and Methods

### Preparation, Partition, and Treatment of AMK Extract

The medical herb AMK (1000 g) was used in this study. The medical herbs were purchased from the Yeongcheon Oriental Herbal Market (Yeongcheon, Korea). All voucher specimens were deposited in the herbal bank of the Korea Medicine (KM)-Based Herbal Drug Research Group, Korea Institute of Oriental Medicine. The total amount of medicinal herbs was placed in 10,000 mL of distilled water and then extracted by heating for 3 hours at 115°C. After extraction, the solution was filtered out using standard testing sieves (150 µm) (Retsch, Haan, Germany), freeze-dried and kept in desiccators at 4°C before use. Stock solution (AMK 20 mg/kg) was filtered using sterilized syringe filter, and was diluted (AMK 0–1000 µg/mL) with media (working solution). Working solution of AMK was variously concentration treated in 3T3-L1 cells. The residue suspended in water and partitioned with Hexan, EtOAc, and BuOH. The fractions were dissolved with dithamdfosufate (DMSO), were filtered using sterilized syringe filter, and were treated at from 0–1000 µg/mL with 3T3-L1 preadipocytes. AA was purchased from Sigma chmical Co. (st. Louis, MO), was treated at from 0–1000 µmol.

### Cell Culture and Adipocyte Differentiation

3T3-L1 cells (ATCC, Manassas, VA) were routinely cultured in growth medium (GM) consisting of DMEM supplemented with 10% FCS (Hyclone, Logan, UT) and 2 mmol glutamine. The cells were differentiated according to a well-established protocol described previously [47]. Briefly, for differentiation, 3T3-L1 cells were cultured in GM to full confluence. Two days after confluence (referred to as day 0), the cells were switched to differentiation media (DM) consisting of DMEM supplemented with 10% FBS, 10 µg/mL insulin, 1 µmol Dex, and 0.5 mmol IBMX and cultured for 3 days. Next, the cells were maintained in DM but containing only insulin (10 µg/mL) and the medium was changed every 2–3 days. The cells normally differentiated into mature adipocytes on day 7 or 8.

### Real Time Polymerase Chain Reaction (RT-PCR)

Total cellular RNA was extracted using TRIzol reagents (Invitrogen, Grand Island, NY) according to instructions provided by the supplier. Quantity and quality of isolated RNA was assessed using nano-drop spectrophotometer (Thermo scientific, Ltd.) and samples were processed for cDNA synthesis using cDNA synthesis kit (Invitrogen. Grans Island, NY). A reaction mixture of 20 µL contained, 2 µg total RNA, 10x RT buffer, dNTP mixture (5 mm each), 10x random hexamer, RNase inhibitor (10 U/µL). The cDNA synthesis was carried out at 37°C for 1 h using a Veriti 96 well thermal cycler (Applied Biosystems, USA). The PCR conditions were as follows: 25 cycles of 94°C for 30 s, 55°C for 30 s, 68°C for 30 s. The amplified products were separated by electrophoresis on 1.5% agarose gels. Primer sequences for PCR analysis were as follow: PPAR-γ (sense) TCACAAGAGGTGACCCAATG, (antisense) CCATCCTTCAC-AAGCATGAA; C/EBP-β (sense) GTTTCGGGAGTTGATGC-AATC, (antisense) AACAACCCCGCAGGAACAT; C/EBP-α (sense) GTGTGCACGTCTA-TGCTAAACCA, (antisense) GCCGTTAGTGAAGAGTCTCAGTTTG; FAS (sense) TGGTGG-GTTTGGTGAATTGTC, (antisense) GCTTGTCCTGCTCTA-ACTGGAAGT; adipocyte fatty acid-binding protein 2 (aP2) (sense) CCAATGAGCAAGTGGCAAGA, (antisense) GATGCC-AGGCTCCAGGATAG; LPL (sense) GGCCAGATTCATCAACTGGAT, (antisense) GCTCCA-AGGCTGTACCCTAAG; adiponectin (sense) GGAGATGCAGGTCTTCTT-GGT, (antisense) TCCTGATACTGGTCGTAGGTGAA; 18s (sense) CATTCGAACGTCTGCCCTATC, (antisense) CCTGCTGCCTTCCTTGGA; β-actin (sense)TGTCCACCTTCCAGCAGATGT, (antisense) AGCTCAGTAACAGTCCGCCTAGA.

### *In vitro* Cytotoxicity Assay

Pre confluent pre-adipocytes (1×10^4^ cells/well) were maintained in 96 well culture plate for 72 h in presence of AMK extract (7.8–2000 µg/mL) of vehicle (PBS). At end of incubation period, 10 µL of MTT (5 mg/ml in PBS4) was added to wells and the plate was incubated at 37°C for 4 h. At the end of incubation, culture media was discarded and the wells were washed with PBS. Later, 150 µL of DMSO was added to all the wells, and were incubated for 30 min at room temperature with constant shaking. Absorbance was read at 540 nm using ELX800 Universal Microplate Reader (Bio-Tek instrucments, Inc., Winooski, VT) and subsequently percentage (%) cell viability was calculated.

### Qualitative Analysis of Adipocyte Differentiation

3T3-L1 pre-adipocytes were differentiated as describe above in presence of AMK extract (100 and 50 µg/mL) or vehicle (PBS). The cells harvested at indicated times during differentiation for stained with Oil-Red O (Sigma, St. Louis, MO) according to the procedure described previously. At the end of incubation (8 days), cells were washed twice with PBS and, fixed in 4% buffered paraformaldehyde solution for 1 h, washed twice DW and then stained with 0.5% Oil-Red O stain for 15 min at room temperature. Excess Oil-Red O dye was washed with DW and photographs were taken in Nikon inverted microscope using Nikon digital camera system. In another set of experiment, the stained adipocytes were treated with 100% isopropanol (to extract intracellular Oil-Red O stain) and the absorbance (Optical density, OD) was read at 520 nm. Percentage adipogenesis was calculated as OD of treated cells/OD of untreated cells×100.

### Ras Activation Assays

3T3-L1 pre-adipocytes were differentiated as described above in presence of AMK extract (100 and 50 µg/mL) and vehicle (PBS) contents were assayed in supernatant and cells respectively. On day 8, after removal of supernatants, cells were washed twice with PBS and, assayed for activation of Ras using commercially Ras activation kit (upstate, Temecula, CA, USA) according to the manufacturer’s instructions.

### Triglyceride Accumulation Assays

3T3-L1 pre-adipocytes were differentiated as described above in presence of AMK extract (100 and 50 µg/mL) or vehicle (PBS) and triglyceride (TG) contents were assayed in supernatant and cells respectively. On day 8, after removal of supernatants, cells were washed twice with PBS and solarized in 100 µL of 1% Triton X-100 and, assayed for total TG using commercially TG-S reaction kit (Asan Pharm. Co., Seoul, Korea) according to the manufacturer’s instructions.

### Protein Extraction and Western Blotting

Cultured and differentiated cells were harvested using a cell scraper and lysed with ice-cold Pro-PREPTM buffer (INtRON, USA). The cell lysates were centrifuged at 14000 rpm for 20 min at 4°C to remove insoluble materials. The protein concentrations were determined using a BCA protein assay kit (Pierce, Rockford, IL). 20 µg of protein extracts was resolved by 10% SDS-polyacrylamide gel electrophoresis and electrotransfered to nitrocellulose membranes at 150 mA for 1 h. The membrane were then blocked for 1 h at room temperature with PBS containing 5% skim milk and 0.1% Tween 20 and incubated with 1∶1000 dilutions of primary antibodies (anti-Akt, anti-pAkt, anti-AMPK, anti-pAMPK, anti-Erk1/2, anti-pErk1/2, anti-PPAR-γ, anti-pMEK1, anti-Raf1 and anti-adiponectin (Cell Signaling, Beverly, MA, USA)) overnight at 4°C and subsequently with a horseradish peroxidase-conjugated anti-rabbit secondary antibody (diluted 1∶1000, Cell Signaling, Beverly, MA) for 1 h at room temperature. Peroxidase activity was visualized using the ECL kit (Thermo, USA).

### High-fat Diet (HFD) - induced Obesity Mouse Model

Purina Diet (Koatech, Seoul, Korea) was provided to mice in the normal diet (ND) group, whereas a pellet rodent diet with 60% Kcal fat (Center Lab. Animal Inc., Seoul, Korea) was provided for 7 weeks to the high-fat diet (HFD) group. Each mouse was administered a dose (62.5 mg/kg) of AMK extract orally. For the vehicle treatment group, the same volume of distilled water was administered orally every day for 7 weeks by the same method used for treatment of AMK extract. Each group comprised eight mice, and all mice were allowed free access to the described diet and water during experimental periods. Body weights and food uptake were measured weekly at regular times.

### H&E Staining of Kidney and Fat Tissue

Fat and kidney tissues were fixed for 48 h in a solution containing 10% formalin. Tissues were dehydrated in ethanol baths (70%, 80%, 90%, and 100%), it’s were embedded in paraffin. Tissues were sectioned at a thickness of 5 µm, and stained with H&E.

### Statistical Analysis

All values in the figures of present study indicate means ± standard deviation (SD), and all determinations were repeated three times. The one way analysis of variance (ANOVA) was used to evaluate the difference among multiple groups, and the independent sample *t* test for difference between two treatment groups. The data were analyzed using GraphPad Prism software (GraphPad Software Inc., Chicago, IL, USA), and *P*<0.05 was assessed as statistically significant.
