# Expression of Concern: *Aristolochia Manshuriensis* Kom Inhibits Adipocyte Differentiation by Regulation of ERK1/2 and Akt Pathway

**DOI:** 10.1371/journal.pone.0355333

**Published:** 2026-08-05

**Authors:** The PLOS One Editors

Following the publication of this article [1], concerns were raised regarding results presented in Fig 4. Specifically, the β-actin panels of Figs 4B, 4C, and 4D appear to partially overlap, calling into question the reliability of these panels and the quantified results associated with these panels.

The authors did not respond to the journal’s queries about these data.

In light of the concerns raised, the *PLOS One* Editors issue this Expression of Concern to notify readers of the above unresolved concerns and to inform readers that the results presented in Fig 4 should be interpreted with caution.
